# MRI-guided personalized tDCS planning for target engagement of the swallowing motor cortex after stroke: a finite-element modeling study

**DOI:** 10.3389/fneur.2026.1898885

**Published:** 2026-09-17

**Authors:** Yeun Jie Yoo, Rak-Kyeun Hong, Mi-Jeong Yoon, TaeYeong Kim, Hyungtaek Kim, Hae-Yeon Park, Sung-Hwa Ko, Hyun Mi Oh, Geun-Young Park, Eun Ji Lee, Tae-Woo Kim, Jisoo Park, Donghyeon Kim, Sun Im

**Affiliations:** 1 Department of Rehabilitation Medicine, St. Vincent’s Hospital, College of Medicine, The Catholic University of Korea, Seoul, Republic of Korea; 2 Research Institute, Neurophet, Inc., Seoul, Republic of Korea; 3 Department of Rehabilitation Medicine, Bucheon St. Mary’s Hospital, College of Medicine, The Catholic University of Korea, Seoul, Republic of Korea; 4 Department of Rehabilitation Medicine, Pusan National University School of Medicine, Pusan National University Yangsan Hospital, Yangsan, Republic of Korea; 5 Department of Rehabilitation Medicine, National Traffic Injury Rehabilitation Hospital, Gyeonggi-do, Republic of Korea; 6 Department of Rehabilitation Medicine, Seoul National University Hospital, Seoul, Republic of Korea; 7 Department of Rehabilitation Medicine, Yeouido St. Mary’s Hospital, College of Medicine, The Catholic University of Korea, Seoul, Republic of Korea; 8 Department of Rehabilitation Medicine, Seoul National University College of Medicine, Seoul, Republic of Korea

**Keywords:** computer simulation, deglutition disorders, electrodes, stroke rehabilitation, transcranial direct current stimulation

## Abstract

**Background:**

Poststroke dysphagia (PSD) is a common, debilitating consequence of stroke that affects up to 50% of patients and leads to multiple medical complications. Transcranial direct current stimulation (tDCS) therapeutic efficacy depends on the electrode montage and interindividual anatomical differences. The use of the standard C5/C6 placement based on 10–20 electroencephalography (EEG) coordinates to guide electrode placements may result in misalignment with the target position.

**Objective:**

To investigate whether computational modeling leads to improved electric field (EF) parameters compared with those generated with standard electrode placement methods, and whether smaller sized electrodes can yield more focal results, we compared EF values across three tDCS montages targeting the cortical swallowing area (CSA): (i) optimized circular (Opt-C, 1.5 cm radius), (ii) EEG-based, conventional circular (Conv-C, 1.5 cm radius), and (iii) conventional square (Conv-S, 5 × 5 cm) electrode montages.

**Methods:**

Structural MRI data from 50 PSD patients were analyzed. EF distributions were simulated using finite element method (FEM) analysis based on three-dimensional tetrahedral head models generated with the tES LAB 4.0 platform. Across montages, we compared EF strength, defined as the mean L2 norm of the EF (E_norm_) across nodes within the target ROI, and target-specific focality, defined as the summed volume of target-ROI elements with E_norm_ values at or above the 75th percentile of the corresponding whole-brain gray and white matter E_norm_ distribution.

**Results:**

Significant differences in EF strength (*p* < 0.001) and target-specific focality (*p* < 0.001) were identified across the three tDCS montages. The Opt-C montage produced the highest EF strength (median: 0.3525 V/m; IQR: 0.306–0.455) and target-specific focality (median: 104.69 mm^3^; IQR: 88.17–120.31). Additionally, small circular electrodes resulted in significantly greater EF strength and target-specific focality than square electrodes (*p <* 0.001).

**Conclusion:**

MRI-guided personalized tDCS planning improved modeled EF delivery to the cortical swallowing target and revealed significant deviations from conventional C5/C6 placement in patients with severe PSD. These findings support image-guided target engagement as a framework for precision neuromodulation in PSD, while prospective physiological and clinical validation is required before therapeutic efficacy can be inferred.

## 1 Introduction

Poststroke dysphagia (PSD) affects approximately 42% of patients in the acute phase of stroke (1) and is often associated with aspiration pneumonia, malnutrition, dehydration, and prolonged hospitalization, which all increase mortality and healthcare costs (2). While traditional interventions, such as dietary modifications and dysphagia rehabilitation remain the standard of care (3), conventional therapies may yield mixed outcomes in some patients who may fail to improve despite intense therapy, highlighting the need for more effective, accessible, and scalable interventions. Enhancing neuroplasticity by noninvasive brain stimulation can lead to improved swallowing performance. Transcranial direct current stimulation (tDCS), by modulating cortical excitability (4), has shown potential to improve outcomes when combined with standard dysphagia therapy and has been suggested as an effective adjunctive treatment for PSD (5). In addition, tDCS is noninvasive and generally well tolerated by even severely affected patients.

Despite the safety, portability, and cost-effectiveness of tDCS, some meta-analyses have suggested that tDCS may have great difficulty in achieving precise stimulation (5, 6) which may limit its clinical efficacy, due to some inherent characteristics. For example, compared with more targeted techniques such as repetitive transcranial magnetic stimulation (rTMS), tDCS generally uses scalp electrodes to pass weak electrical currents through the head, aiming to modulate brain activity in specific regions. However, the electric fields (EFs) generated are widespread (i.e., less spatially focused), which can extend well beyond the intended target area. One contributing factor may be the use of large electrodes (measuring 5 × 5 cm or 5 × 7 cm), which generate diffuse EFs with poor focality, potentially reducing the precision of cortical targeting (7, 8). Moreover, the effectiveness of tDCS is significantly influenced by interindividual differences in the relevant anatomy, including skull thickness, scalp conductivity, brain morphology, and lesion characteristics (9), limiting the ability to consistently deliver the current to the target area within the motor cortex.

While these concerns affect all uses of tDCS, they are particularly critical in PSD. The cortical swallowing area (CSA) is located within the primary motor cortex (M1), with marked interindividual differences in location and hemispheric lateralization (10). Using single-pulse TMS, the CSA has been shown to be localized in the lateral pericentral cortex, showing an oro–pharyngo–esophageal somatotopic gradient that shifts from more lateral-anterior (mylohyoid) to more medial-posterior (esophagus), corresponding to a scalp area of roughly 1.5–2.0 cm in diameter (11). The CSA’s proximity to functionally distinct regions, such as Broca’s area and the insular cortex, raises the possibility of unintended stimulation of adjacent regions with conventional electrode montages. Therefore, reliance on conventional 10–20 electroencephalography (EEG) coordinates to guide electrode placements may direct peak EFs toward adjacent motor areas not involved in swallowing (12). Finally, interindividual differences in anatomical characteristics such as skull thickness, particularly near the temporal bone close to the CSA, can result in significant variability in the delivered EF strength (13). Among these anatomical factors, cerebrospinal fluid (CSF) is particularly influential: because its conductivity is far higher than that of skull and scalp, CSF acts as a low-resistance path that shunts and widely redistributes current across the cortical surface, making its distribution a primary determinant of current spread and interindividual variability (14, 15). These limitations motivate the development of advanced strategies to optimize tDCS delivery to swallowing-related targets while minimizing off-target effects and interindividual variability. We therefore sought to determine whether MRI-guided individualized anode placement could increase modeled EF strength and target-specific focality within the intended swallowing motor target compared with conventional fixed-coordinate montages.

Previously, TMS-guided motor-evoked potentials (MEPs) have been used to guide electrode configurations in the development of tDCS schemes, allowing for anodes to be positioned directly over the optimal site for inducing motor responses (10). However, this approach has several limitations. This method requires the use of specialized equipment and trained operators and can be semi-invasive, particularly when it involves the recording of pharyngeal MEPs with intraluminal catheter electrodes, resulting in patient discomfort and limiting its routine application in clinical environments. Furthermore, cortical excitability may be reduced or absent in stroke patients. In addition, MEP mapping provides surface-level functional hotspots that may not correspond precisely with the anatomical sites of interest, particularly in sulcal regions (16). Moreover, MEPs are obtained from either the mylohyoid or pharyngeal level, but swallowing requires the activation of both locations. These challenges highlight the need for a noninvasive, reproducible, and automatic method that does not require MEP localization to guide electrode placement and can increase focality and EF strength when targeting the swallowing motor cortex.

One approach to addressing these limitations is computational modeling, which enables patient-specific prediction of the EF distribution. By accounting for the brain and skull anatomy of the patient, computational modeling allows the optimization of electrode montages with stronger and more focal EF distributions, potentially guiding more personalized treatment than conventional “one-size-fits-all” approaches. Specifically, modification of the electrode configuration, such as via adjustment of the size and shape of the electrodes or interelectrode distance, may enable more precise targeting and the generation of a more focal EF than what can be achieved with conventional electrode montages (13).

Importantly, stroke-related anatomical alterations may substantially modify current flow patterns during tDCS. Prior in silico studies have optimized electrode placement in other stroke/neurological targets (aphasia, hand paresis, gait); the present study extends this to the anatomically distinct and variable swallowing motor representation, not previously addressed (17–19). In our previous work (9), we demonstrated that lesion location can significantly alter the spatial distribution of the induced EF, leading to considerable variability in stimulation intensity at the intended cortical targets. These findings suggest that conventional electrode placement based on the standard 10–20 EEG system may fail to deliver optimal stimulation in stroke populations. Therefore, individualized electrode positioning based on patient-specific anatomical information may be essential for achieving reliable neuromodulatory effects. However, how to implement such individualized targeting in PSD remains unexplored.

Accordingly, we employed tES LAB 4.0 (Neurophet Inc.), a simulation platform that integrates deep neural network–based stroke lesion segmentation into individualized head models, thereby enabling EF estimation that accounts for the altered electrical conductivity of affected tissue (20). Furthermore, we adopted a target-specific, region-of-interest (ROI)–based focality metric tailored to the CSA, rather than relying on conventional whole-brain focality indices that may inadequately capture stimulation precision within small, functionally defined cortical targets.

Therefore, the present in silico study was designed to address three unresolved issues in tDCS for PSD. First, we examined whether stroke-specific MRI-based computational modeling, incorporating individualized lesion segmentation, can improve EF targeting beyond that achieved with conventional 10–20 EEG coordinate–based electrode placement. Second, we investigated whether small circular electrodes could enhance stimulation precision compared with conventional large square electrodes. Third, we evaluated whether a target-specific focality metric—quantifying EF concentration within a predefined swallowing motor ROI—provides a more clinically relevant assessment of stimulation precision than global EF spread indices. Using individualized MRI-derived head models generated with the tES LAB 4.0 platform and a grid-search–based optimization algorithm, we compared three tDCS montages: the optimized circular (Opt-C), conventional circular (Conv-C), and conventional square (Conv-S) montages. We hypothesized that a computationally optimized montage employing small circular electrodes would produce both stronger and more focal EFs within the swallowing motor ROI than conventional montages based on fixed scalp coordinates. The clinical translation of this modeling framework is being evaluated prospectively in a multicenter, double-blind, sham-controlled randomized trial (NCT06305949) that enrolled 30 patients with PSD and used the same MRI-guided individualized targeting protocol and 1.5-cm-radius circular electrode geometry evaluated in the present study (21). Participant enrollment has been completed, and analyses of the swallowing outcomes are underway.

## 2 Methods

### 2.1 Study design and participants

This multicenter, retrospective *in silico* study included MR imaging data from 50 patients with confirmed PSD acquired from January 1, 2013, to July 31, 2023. The patient inclusion criteria were as follows: age 19 years or older at the time of MRI; diagnosis of first-ever ischemic stroke with no evidence of intracerebral hemorrhage; and dysphagia severe enough to require tube feeding, as confirmed by a videofluoroscopic swallowing study (VFSS). The exclusion criteria were as follows: any space-occupying or intracranial tumors, hemorrhagic transformation within the infarcted area, and poor image quality that impaired modeling or imaging data containing personally identifiable patient information.

### 2.2 Ethical approval

The study protocol was approved by the Institutional Review Boards (IRBs) of the Catholic University College of Medicine (approval number: XC23DSDS0090), Pusan National University School of Medicine (approval number: 2023-09-001), and the National Traffic Injury Rehabilitation Hospital (approval number: 22-2023-001), Republic of Korea. All procedures were conducted in accordance with the Declaration of Helsinki, and the need for written informed consent was waived due to the retrospective nature of the study.

### 2.3 tDCS simulation pipeline

All tDCS simulations were performed using tES LAB 4.0, an integrated simulation platform designed for patient-specific EF modeling. A key feature distinguishing tES LAB 4.0 from conventional simulation tools is its ability to incorporate stroke lesion segmentation directly into the head modeling pipeline, enabling accurate representation of the altered electrical conductivity of affected tissue. This capability is essential for generating biophysically realistic EF distributions in stroke populations, where lesion-related tissue changes can substantially affect current flow patterns. We simulated tDCS for each patient via a three-step process: MRI segmentation, anatomical modeling, and EF estimation (Figure 1).

**Figure 1:**
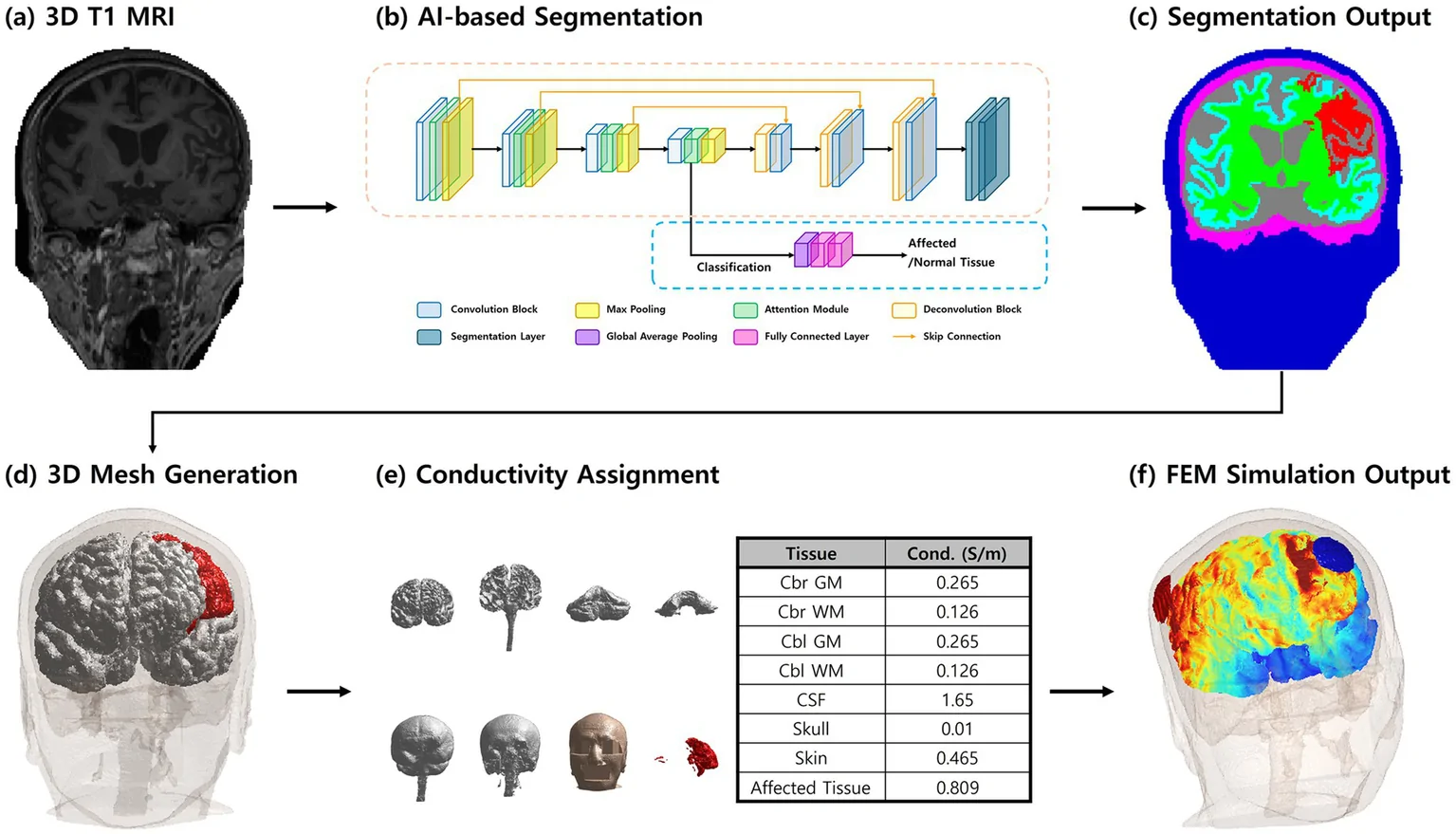
Brain segmentation and tDCS simulation process using tES LAB 4.0. **(a)** The tDCS simulation process begins with importing a high-resolution 3D T1-weighted MRI scan into tES LAB 4.0 software. **(b)** An AI-based segmentation model automatically classifies head tissues **(c)**, including affected regions, for subsequent use in anatomical modeling. **(d)** A 3D tetrahedral mesh is generated on the basis of the segmented image. **(e)** Conductivity values are assigned to individual mesh elements according to the physical properties of their corresponding tissue types. **(f)** A finite element method (FEM) simulation is subsequently performed to compute the distribution of the electric field across the brain. FEM, finite element method; tDCS, transcranial direct current stimulation.

#### 2.3.1 MRI segmentation step

For the MRI segmentation step, we used tES LAB 4.0’s built-in deep neural network segmentation module, which segments MR images into distinct layers and is effective for creating head models of stroke patients by accurately identifying and segmenting affected tissues. A previous study (20) has demonstrated its superior performance over conventional segmentation models such as U-Net, U-Net++, HighResNet, and DeepLab V3 (22, 23). The required structural input was a high-resolution three-dimensional T1-weighted volume with whole-brain coverage and image quality sufficient for tissue segmentation and head-model construction. Manufacturer-specific acquisition parameters recommended for tES LAB 4.0 are summarized in Supplementary Table S1. All included images met these core input and image-quality requirements.

#### 2.3.2 Anatomical modeling

For the anatomical modeling step, we created a three-dimensional tetrahedral mesh using the Delaunay triangulation method in the Computational Geometry Algorithms Library (CGAL) (24). We densely distributed the nodes in the skin layer to accurately represent the placement of surface electrodes directly on the skin surface. Accordingly, the electrodes and conductive media, such as gel or paste, were not explicitly modeled.

#### 2.3.3 EF estimation

To estimate EF, we discretized the tDCS problem using the finite element method (FEM). On the basis of data from the literature, isotropic electrical conductivity values were assigned to each tissue layer: the cerebral gray matter (GM; 0.265 S/m), cerebral white matter (WM; 0.126 S/m), cerebellar GM (0.265 S/m), cerebellar WM (0.126 S/m), cerebrospinal fluid (CSF; 1.65 S/m), skull (0.01 S/m), skin (0.465 S/m), and affected tissues (0.809 S/m) (25–27). In the quasistatic regime, where the time-varying electromagnetic fields are negligible, Maxwell’s equations reduce to the Laplace (Equation 1):

∇·(σ∇V)=0inΩ (1)

where V is the tDCS-induced potential and Ω is the anatomical head model. The insulation condition at the outer boundaries is satisfied when the inner product of the normal vector (*n*) and the current density is zero, expressed as the Neumann boundary condition in Equation 2:

σ∇V⋅n=0 (2)

where σ is the electrical conductivity of the tissue layer in question. We then computed the EFs using the conjugate gradient (CG) method with the Eigen library (28), setting the termination criterion at 10^−18^. Subsequently, the EFs within the tetrahedra were derived by calculating the gradient of the potential V, which was linearly interpolated from the nodal values using first-order Lagrange elements. These fields were then transformed into a nodewise format via linear interpolation, defining the directional EF components ( $E_{x}$ , $E_{y}$ , $E_{z}$ ) at each node.

Before the simulations were started, the target CSA was localized to the precentral gyrus on the basis of convergent evidence from task-based functional magnetic resonance imaging (fMRI) investigations of volitional swallowing (29, 30). Given the well-documented somatotopic organization of the cortical representation of swallowing, with lateral regions corresponding to mylohyoid function (important for oral phase control) and more medial zones associated with pharyngeal control (11, 31, 32), we chose to position the initial tDCS target at the center point between these functional sites. This midpoint position was chosen to represent both oral- and pharyngeal-related subregions within the primary swallowing motor cortex rather than either subregion alone. The target was transformed into the anatomical spaces of each patient via rigid registration of their high-resolution T1-weighted MR images (1 mm^3^ isotropic). The target was consequently operationalized as a spherical region of interest (ROI) with a 2-mm radius; this size was selected to maintain computational feasibility while preserving anatomical specificity. The resulting individualized target served as the primary means for assessing EF optimization, as it allowed quantitative assessments of EF strength and target-specific focality across the candidate electrode configurations. To ensure accurate and clinically meaningful targeting of the CSA, a clinician with expertise in neuroimaging and PSD identified and validated the hotspot from data derived from the literature on fMRI data associated with swallowing.

EF strength was quantified as the L2-norm of its directional components (E_norm_), representing the magnitude of the induced EF at each node within the target region. To assess target-specific focality, we quantified the summed volume (mm^3^) of tetrahedral elements within the predefined swallowing motor ROI (V_ROI_) whose nodal EF strength was greater than or equal to the 75th percentile (P_75_) of EF strength values computed across all GM and WM nodes of the head model. This volumetric computation is analogous to conventional whole-brain focality method that sum suprathreshold tetrahedral element volumes (33). Because the ROI is defined by all tetrahedral elements connected to nodes falling within the 2-mm-radius target sphere, the effective ROI volume exceeds the geometric volume of the sphere itself, reflecting the spatial extent of the constituent mesh elements, as expressed in Equation 3:

$$\text{Target specific focality}\;(mm^{3})=V_{\mathrm{ROI}}|E_{\mathrm{norm}}\geq P_{75}$$ (3)

This target-specific focality metric differs fundamentally from conventional whole-brain measures, which assess the overall volume of highly stimulated tissue regardless of anatomical specificity. By restricting the analysis to a clinically defined swallowing motor region, this approach directly quantifies how much of the intended therapeutic target receives high-intensity stimulation—providing a more physiologically and clinically meaningful index of precision than global EF spread metrics. Increased target-specific focality under this definition therefore indicates a greater concentration of stimulation within the swallowing motor cortex.

### 2.4 Electrode configuration

We assessed two types of electrodes: a circular electrode (radius = 1.5 cm) and a square electrode (5 × 5 cm). Previous studies have shown that small electrodes (e.g., of area 3.5 cm^2^) can modulate excitability in the target region without affecting adjacent areas (34) and that 1 cm × 1 cm electrodes produce the strongest and most focal EFs (35). Accordingly, we compared the EF strengths produced by three tDCS montages: (i) Opt-C, (ii) Conv-C, and (iii) Conv-S.

In the conventional tDCS montage, the electrodes were placed in accordance with the 10–20 EEG system positions (36). Specifically, the anode was placed over CSA at C5 or C6 (midpoint between C3-T3 or C4-T4, respectively by the 10–10 EEG system) in the unaffected hemisphere, with the cathode positioned over the supraorbital cortex (AF3 or AF4) in the affected hemisphere (Figure 2). Moreover, two simulation conditions were tested, involving the same electrode positions but different electrode shapes (square vs. circular). In both simulations, a direct current of 2 mA was applied through the anode. This intensity has been demonstrated to be safe and clinically effective in PSD (37).

**Figure 2:**
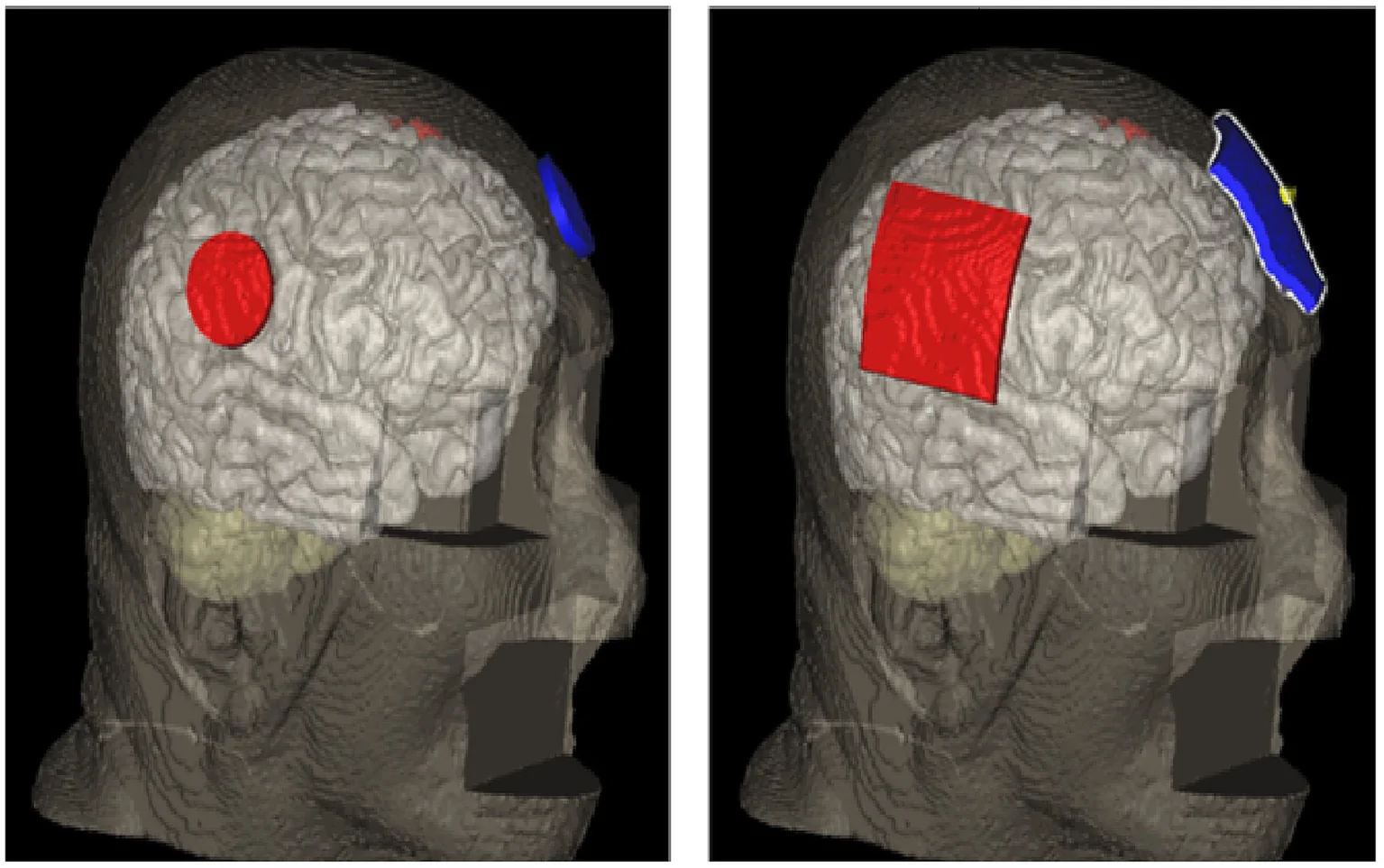
Electrode and montage configurations. Left: Circular electrode (radius = 1.5 cm) used in both the optimized (Opt-C) and conventional circular (Conv-C) montages. Right: Square electrode (5 × 5 cm) used in the conventional square (Conv-S) montages. For both conventional montages, the anode (red) was placed over the unaffected hemisphere at C5 or C6 (midpoint between C3-T3 or C4-T4, respectively, according to the 10–10 EEG system). For all montages, the cathode (blue) was placed over the affected hemisphere (at AF3 or AF4). EEG, electroencephalography.

For the optimized tDCS electrode montage, we implemented circular electrodes with a radius of 1.5 cm to deliver a direct current of 2 mA, identical to that used for the conventional montages. We first used a grid search algorithm to generate candidate electrode positions in a grid array near the initial location and determine the optimal electrode placement. The search space fully encompassed the motor hotspot associated with swallowing functions, as identified by prior EEG experiments (38), with one of the midpoints between C3-T3 and C4-T4 set as the initial reference point (39). We iteratively evaluated different candidate montages to identify the montage that resulted in a maximized EF within the functional target of the ROI.

We chose to place the anodes on the unaffected hemisphere based on prior evidence from both experimental (40) and clinical tDCS studies (41) indicating that poststroke swallowing recovery occurs due to neuroplasticity driven by the intact motor cortex.

### 2.5 Electrode displacement and stroke lesion volume calculation

Electrode displacement analysis quantifies how optimized montages differed spatially from conventional montages, with larger displacements indicating that optimization differs substantially from standard placement, indicating the need for personalization. To quantify electrode displacement, we calculated the interelectrode distance (D), defined as the three- dimensional Euclidean distance between center positions of the conventional and optimized electrode positions (9). Since only the placement of the anode was optimized while the position of the cathode remained fixed, the cathode interelectrode distance (D_cathode_) was 0, and consequently, the anode interelectrode distance (D_anode_) was equal to D. Additionally, for each montage condition, we calculated the distance between the centers of the anode and target ROI (D_anode-target_) and the within-montage interelectrode distance between the centers of the anode and cathode (D_anode-cathode_).

In addition, stroke lesion volume analysis provides data on how affected tissue extent affects EF distribution patterns and individual treatment responses. The stroke lesion volume was determined by calculating the voxel-based volume of the affected brain tissue, as segmented from the MR images.

### 2.6 Data and statistical analysis

Calculations of the EF strength and target-specific focality within the target ROI, electrode placement, and visualization of the EF distribution were performed using MATLAB (version 2024a; MathWorks Inc., Natick, MA, USA). Statistical analyses and visualizations were conducted using R software (version 4.4.0; R Core Team). All statistical tests were two-sided, with statistical significance set at α = 0.05.

For all data to be subject to paired comparison analysis, normality was assessed with the Shapiro–Wilk test, and sphericity was evaluated with Mauchly’s test. None of the data regarding EF strength or target-specific focality followed a normal distribution (EF strength: *p <* 0.05; target-specific focality: *p <* 0.001). Moreover, neither the EF strength nor target-specific focality satisfied the assumption of sphericity based on the results of Mauchly’s test (EF strength: *χ^2^*(2) = 52.769, *p <* 0.001; target-specific focality: *χ^2^*(2) = 124.279, *p <* 0.001).

Owing to the violations of normality and sphericity, we used the Friedman test, a nonparametric alternative, to assess differences in EF strength and focality among the three configurations. *Post hoc* analyses were performed with the Wilcoxon signed-rank test. To control Type I errors from multiple comparisons, the *p* values were adjusted with Bonferroni correction.

To confirm that the montage comparison was not driven by heterogeneity in lesion characteristics, we repeated the primary analyses in a linear mixed-effects framework with montage as a within-subject factor and a random intercept per subject, fitted by restricted maximum likelihood. Lesion volume (z-standardized), lesion side and lesion location (cortical, subcortical or combined) were entered as between-subject predictors, and montage × lesion interactions were tested. Two prespecified sensitivity analyses were performed: (i) lesion location recoded as presence versus absence of cortical involvement, on the anatomical rationale that cortical GM involvement alters local conductivity and the cortical current-sink geometry in a way that subcortical white-matter injury does not, and (ii) exclusion of the three patients with combined cortical and subcortical lesions.

To examine how D and stroke lesion volume are related to changes in EF strength and target-specific focality, we performed Spearman’s correlation analyses. Changes in EF strength and target-specific focality were calculated as the differences between the optimized and circular montages. To distinguish improved anode-target alignment from altered current-path geometry, D_anode-target_ and D_anode-cathode_ were compared between montages using Wilcoxon signed-rank tests, with matched-pairs rank-biserial correlations reported as effect sizes. Changes in D_anode-target_ and D_anode-cathode_ were subsequently entered simultaneously into separate linear models for changes in EF strength and target-specific focality to estimate their independent associations with each outcome.

## 3 Results

### 3.1 Descriptive patient characteristics

Among the 50 patients whose MRI data were included in the study, 27 (54.0%) were male, and the mean age was 73.6 ± 10.7 years. Additionally, the lesions of 31 of the 50 patients (62.0%) were in the left hemisphere (Table 1). All patients had confirmed severe dysphagia that required tube feeding at the time of MRI acquisition verified by medical records.

**Table 1:** Demographic and clinical characteristics of participants.

Variable	Value
Age (years)	73.6 ± 10.7
Lesion volume (cm^3^)	54.3 ± 58.8
Lesion side	Right hemisphere: 19 (38%)
	Left hemisphere: 31 (62%)
Sex	Male: 27 (54%)
	Female: 23 (46%)
Lesion location	Cortical: 19 (38%)
	Subcortical: 28 (56%)
	Combined: 3 (6%)

Values are presented as mean ± standard deviation (SD) for continuous variables and as number (percentage) for categorical variables.

### 3.2 Comparison of EF strength and target-specific focality across montages

To assess the impact of electrode montage design on the strength and concentration of the EF, we compared the EF strength and target-specific focality resulting from the simulated use of three tDCS montages. Anode locations across all patients and montage conditions are shown in Supplementary Figure S1. According to the nonparametric Friedman test, both the EF strength [χ^2^(2) = 100, *p <* 0.001] and focality [χ^2^(2) = 53.47, *p <* 0.001] differed significantly among the three montages. Among the three montages, the Opt-C montage produced the highest median EF strength (0.3525 V/m, IQR: 0.3063–0.4553 V/m), followed by the Conv-C (0.3240 V/m, IQR: 0.2741–0.4037 V/m) and Conv-S (0.2982 V/m, IQR: 0.2523–0.3558 V/m) montages (Figure 3a). Pairwise Wilcoxon signed-rank tests with Bonferroni correction confirmed that all intermontage differences were statistically significant (all *p*_bonf_ < 0.001). Specifically, the Opt-C montage produced a median EF increase of 0.0285 V/m (8.8%) relative to Conv-C and 0.0543 V/m (18.2%) relative to Conv-S, indicating that the Opt-C montage achieves significantly stronger EF at the target compared to conventional montages.

**Figure 3:**
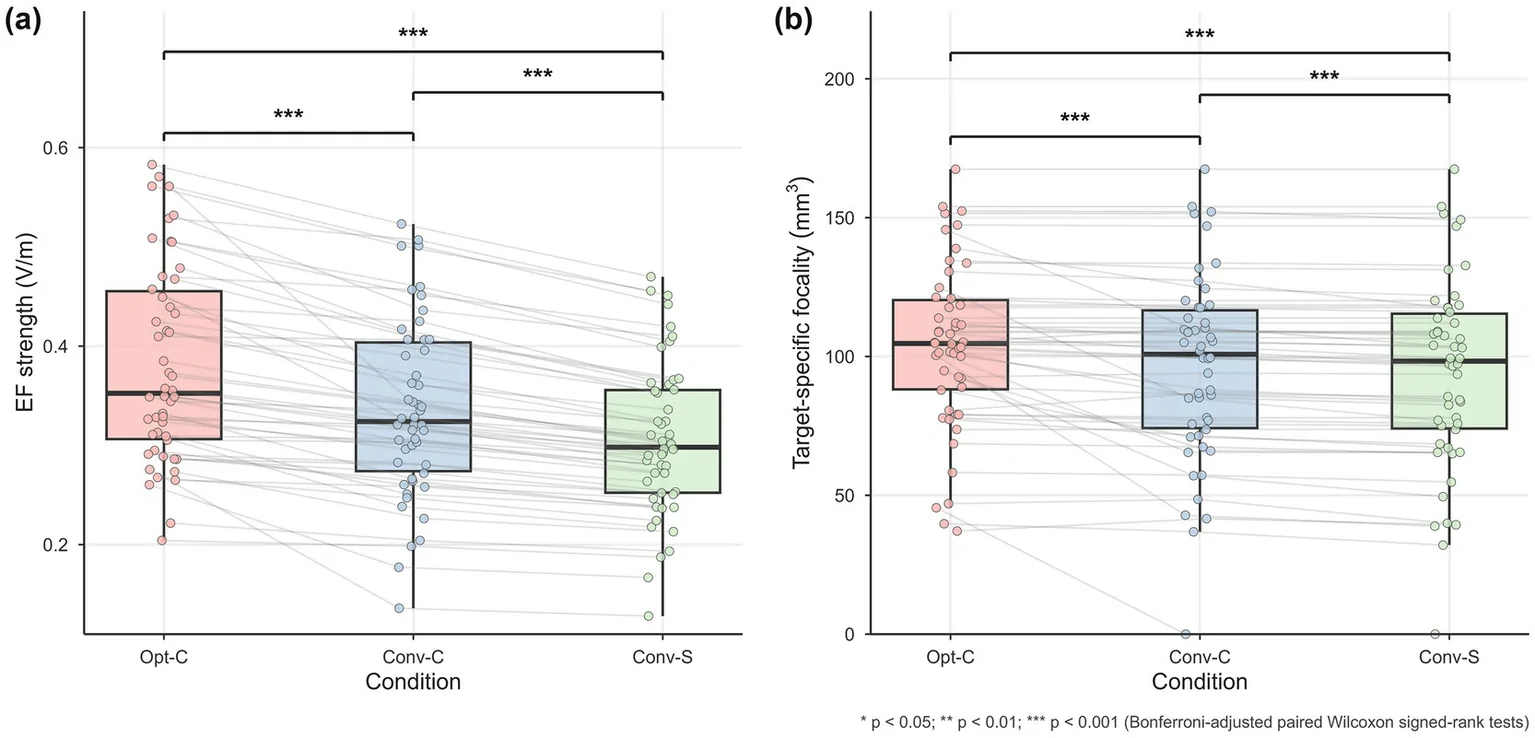
Comparison of EF strength and target-specific focality across electrode montages. **(a)** EF strength and **(b)** target-specific focality. Individual patient values are shown as jittered points, with lines connecting measurements from the same patient across the three montage conditions. The box represents the interquartile range (IQR) with the median line. Significant differences were observed among conditions (*p* < 0.001). The montages are labeled as follows: Opt-C (optimized circular, 1.5 cm radius), Conv-C (conventional circular, 1.5 cm radius), and Conv-S (conventional square, 5 × 5 cm). *p < 0.05, **p < 0.01, ***p < 0.001. EF, electric field.

Similarly, target-specific focality was highest in the Opt-C montage (median: 104.69 mm^3^, IQR: 88.17–120.31 mm^3^), followed by the Conv-C montage (100.80 mm^3^, IQR: 74.20–116.62 mm^3^), and lowest in Conv-S (98.31 mm^3^, IQR: 74.06–115.40 mm^3^) (Figure 3b). Similar to the EF strength, all paired differences in target-specific focality were statistically significant (*p*_bonf_ < 0.001). Thus, the Opt-C montage resulted in significantly greater target-specific focality than the conventional montages, and the Conv-C montage achieved greater target-specific focality than the Conv-S montage. Circular electrodes consistently generated a stronger EF in all cases, whereas greater target-specific focality was observed with the use of circular electrodes for 72% (36/50) of the patients. In contrast, there were no instances where the square electrode demonstrated superior target-specific focality. These results indicate that Opt-C montage produces significantly greater target-specific focality alongside enhanced EF strength, compared to conventional electrode montages.

In mixed-effects models simultaneously adjusting for lesion volume, lesion side, and lesion location, the advantage of the optimized montage remained unchanged. Compared with Conv-C, Opt-C produced a higher EF strength by an adjusted mean difference of 0.0447 V/m (95% CI, 0.0351–0.0544; *p* < 0.001) and greater target-specific focality by 7.44 mm^3^ (95% CI, 4.08–10.80; *p* < 0.001). Neither lesion volume nor lesion side was associated with EF strength (*p* = 0.298 and *p* = 0.670, respectively), whereas larger lesion volume was associated with greater target-specific focality (*β* = 13.36 mm^3^ per 1 SD; *p* = 0.023). There was no evidence that the montage effect varied according to lesion volume, lesion side, or lesion location for either outcome. Joint Wald tests for the montage-by-lesion-volume interaction were non-significant for EF strength (*p* = 0.547) and target-specific focality (*p* = 0.843), as were the montage-by-lesion-side interaction (*p* = 0.947 and *p* = 0.763, respectively) and the montage-by-lesion-location interaction (*p* = 0.640 and *p* = 0.923, respectively). The findings were unchanged when lesion location was recorded according to the presence or absence of cortical involvement; the corresponding interaction was non-significant for both EF strength (*p* = 0.606) and target-specific focality (*p* = 0.888). After excluding the three patients with combined cortical and subcortical lesions, the adjusted Opt-C versus Conv-C difference remained 0.0440 V/m for EF strength (95% CI, 0.0339–0.0542; *p* < 0.001). At the individual level, Opt-C produced a higher EF strength than Conv-C in all 28 patients with subcortical lesions, all 19 patients with cortical lesions, and all three patients with combined lesions.

### 3.3 Correlation analysis of the electrode displacement and stimulation parameters

The relationship between electrode displacement and stimulation efficacy was examined through correlation analysis involving the EF distributions across three tDCS montages. The displacement between the optimized and conventional electrode positions reached a maximum of 36.5 mm and a median of 20.6 mm (IQR: 15.1–27.2 mm). D was positively correlated with changes in both EF strength (*r_s_* = 0.651, *p <* 0.001; Figure 4a) and target-specific focality (*r_s_* = 0.526, *p <* 0.001; Figure 4b). In contrast, lesion volume was not significantly correlated with change in EF strength (*r_s_* = −0.173, *p* = 0.229), the corresponding change in target-specific focality, defined as suprathreshold target volume (*r_s_* = 0.096, *p* = 0.508), or D (*r_s_* = −0.122, *p* = 0.399). These findings suggest that D is positively associated with improvements in EF characteristics, supporting the advantage of individualized electrode positioning in the Opt-C montage over conventional approaches.

**Figure 4:**
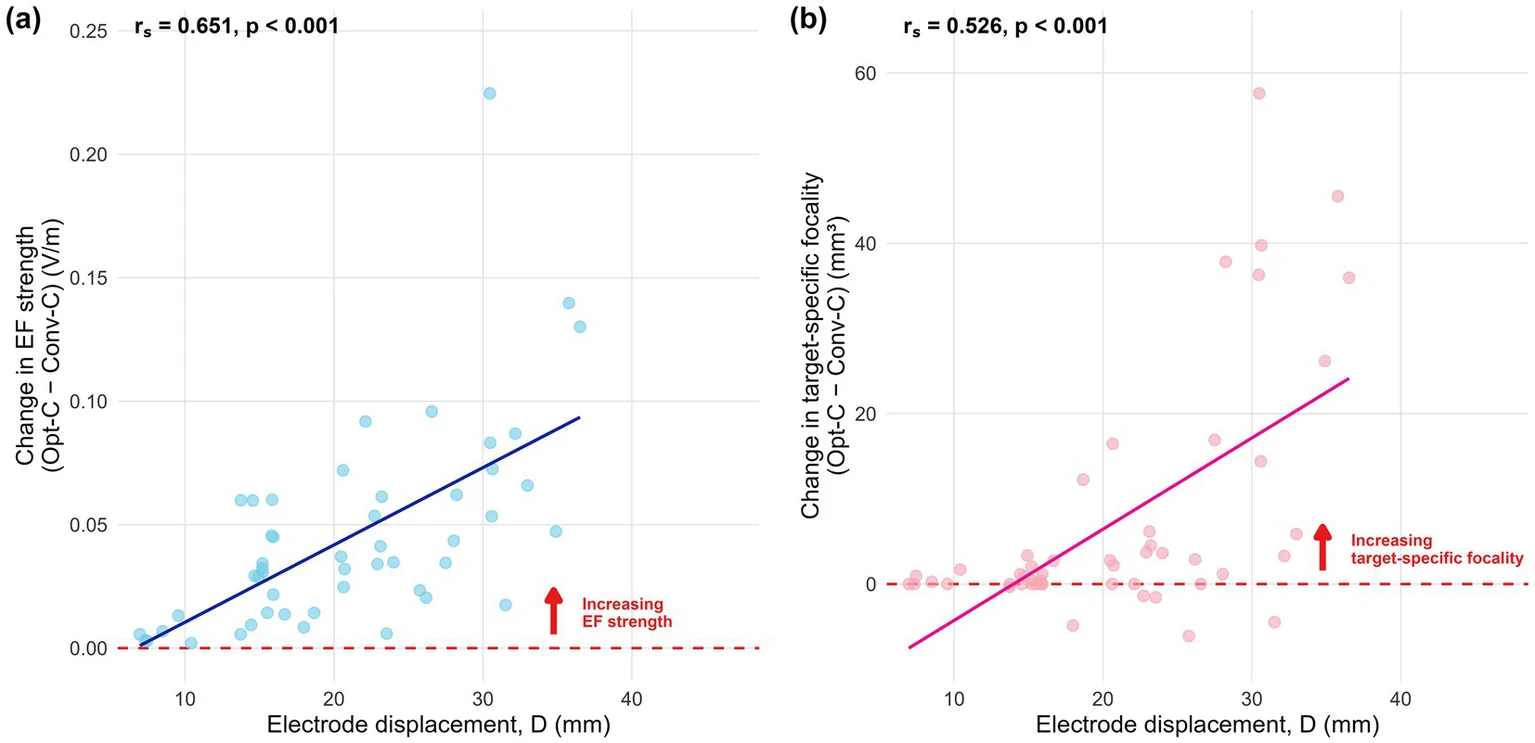
Correlations of electrode displacement (*D*) with changes in *EF* **(a)** and target-specific focality **(b)**. Red dashed line indicates no change in *EF* and target-specific focality. Positive correlations between *D* and both *EF* and target-specific focality were found. *EF*, electric field; *D*, electrode displacement.

The optimized anode was located significantly closer to the individualized cortical target than the conventional anode (median 33.9 mm, IQR 31.2–37.4 vs. 37.5 mm, IQR 34.4–40.4; Wilcoxon signed-rank *p* < 0.001; matched-pairs rank-biserial correlation, *r_rb_* = −0.545), and was closer in 37 of 50 patients (74%). The D_anode-cathode_ was also shorter for the optimized montage (median 124.8 vs. 134.0 mm; *p* < 0.001; *r_rb_* = −0.561), indicating that the change in target EF reflects both improved target proximity and altered current-path geometry. The magnitude of the EF gain was not fully explained by target-approach distance alone (*r_s_* = 0.17, *p* = 0.23). The reduction in D_anode-target_ was not significantly associated with the gain in target EF strength (*r_s_* = 0.172, *p* = 0.232). In contrast, the reduction in D_anode-cathode_ showed a moderate positive association with EF gain (*r_s_* = 0.444, *p* = 0.0012). In 13 patients, the optimized anode was located farther from the individualized target than the conventional anode. Nevertheless, target EF strength increased in all 13 patients. The magnitude of EF gain did not differ significantly between patients whose optimized anode moved farther from the target and those whose optimized anode moved closer to the target: 0.032 V/m (IQR: 0.007–0.066 V/m) versus 0.035 V/m (IQR: 0.020–0.060 V/m), respectively (Mann–Whitney U = 225, *p* = 0.740). Overall, target EF strength increased under the optimized montage in all 50 patients.

## 4 Discussion

The present study is a target-engagement modeling study, not a clinical efficacy trial; its purpose was to determine whether MRI-guided electrode optimization could increase EF delivery to the swallowing motor cortex beyond that achieved with fixed-coordinate placement. Our results demonstrate that the optimized montage (Opt-C) produced significantly stronger EFs at the CSA target than conventional montages in 50 PSD patients. In our optimization method, the supraorbital cathode was kept positionally fixed. This deliberate constraint allowed us to isolate anode position as the only searched variable within a standardized return-electrode configuration. However, anode repositioning altered both the anode-to-target distance and anode-to-cathode geometry; therefore, the observed EF differences represent the overall geometrical effect of anode repositioning on the current path rather than target approximation alone. The correlation between electrode displacement and EF gain indicates that personalization is likely most beneficial when the swallowing-related target is anatomically misaligned with standard C5/C6 placement. This finding implies that a fixed C5/C6 montage may result in target underdosing in a subset of patients, and that individualized modeling can identify and correct such misalignment. These results provide the first computational evidence supporting MRI-based personalized electrode optimization for PSD, establishing a biophysical rationale for subsequent clinical validation.

The clinical significance of these findings is particularly evident in the context of severe PSD, where the functional state of the corticobulbar pathway may be substantially compromised, further amplifying interindividual variability in optimal stimulation sites—variability that fixed-coordinate placement cannot adequately address. Moreover, although MEP-guided targeting can improve anatomical precision, its dependence on specialized pharyngeal recording equipment and technical expertise limits its applicability in routine clinical settings.

Our MRI-based optimization approach offers a noninvasive alternative for individualized targeting. By using only structural MRI data, our method identified optimized electrode positions that deliver stronger EFs to the swallowing motor target without requiring invasive physiological mapping. This suggests a potentially alternative pathway toward individualized neuromodulation in PSD that warrants prospective clinical validation.

Although computationally guided tDCS optimization has been investigated in other neurological conditions, including poststroke aphasia (17), hand paresis (18), and gait disturbances (19), the present application to PSD has distinct significance. Swallowing is supported by a relatively compact and anatomically variable cortical representation, and successful stimulation requires engagement of both oral- and pharyngeal-related motor subregions. Therefore, the technical demands of targeting the swallowing motor system may be greater than those for more commonly studied motor areas. Our findings suggest that the combination of individualized electrode positioning and smaller electrode geometry is particularly well suited to these constraints and may represent a more practical strategy for improving on-target EF delivery in PSD than conventional fixed-position montages.

Our computational approach targeted the CSA using anatomical landmarks from published fMRI studies, allowing the identification of individualized electrode positions that produce stronger EFs than conventional fixed-coordinate methods while eliminating the need for invasive MEP-based localization. Using only structural MRI scans, the optimized montages achieved 8.8% greater median target EF than conventional circular and 18.2% greater than conventional square electrodes. These results align with recent optimization studies in other stroke populations (9, 18), which showed improved EF targeting in hand motor cortex; however, targeting the CSA represents distinct challenges, as the swallowing motor region is anatomically variable, lies more lateral than other motor representations, and requires simultaneous engagement of both mylohyoid and pharyngeal subregions. The present study is the first to apply computational optimization to address the targeting constraints specific to the swallowing motor system.

In addition to electrode positioning, electrode geometry contributed to improved on-target EF delivery. In the present comparison, the smaller circular electrodes (area ≈ 7.07 cm^2^) consistently produced stronger EFs and greater target-specific focality than the larger square electrodes (area = 25 cm^2^). It should be noted that this comparison confounds two variables—electrode size and electrode shape—because the circular electrodes were both smaller in area and different in geometry from the square electrodes. Nevertheless, the observed advantage is consistent with previous computational work demonstrating that reducing electrode area enhances field concentration at the target (34), whereas larger electrode areas produce more diffuse current distributions (42, 43). In addition, computational studies have shown that circular geometries minimize the edge-effect current accumulation that occurs at the corners of rectangular electrodes, potentially contributing to a more uniform current distribution beneath the electrode (44). Thus, the improved on-target delivery observed with circular electrodes in this study likely reflects the combined effect of reduced electrode area and the absence of corner-related current inhomogeneities.

Our results support a dual-optimization strategy for PSD: personalization of electrode location based on individual anatomy, combined with the use of smaller circular electrodes. This combined approach may offer advantages over conventional tDCS montages that rely on large rectangular electrodes and fixed scalp landmarks. This strategy also differs from conventional high-definition tDCS (HD-tDCS) while sharing the goal of improved spatial precision. HD-tDCS systems using the 4 × 1 configuration deploy multiple small electrodes around a central electrode to achieve high focality (45), but their effectiveness is highly sensitive to interindividual anatomical differences, which may complicate clinical implementation (46). In contrast, our approach achieves meaningful target-specific focality improvements using only two electrodes with computationally optimized placement, maintaining the operational simplicity of standard tDCS while providing a level of anatomical precision that fixed-coordinate montages cannot offer. This simplicity may favor adoption in clinical settings where multielectrode setups are challenging to implement.

The positive correlation between electrode displacement and EF gain (*r_s_* = 0.651 for EF strength; *r_s_* = 0.526 for target-specific focality) suggests that the benefit of personalized optimization may be greatest in patients whose swallowing-related cortical target is anatomically misaligned with standard C5/C6 placement. The median displacement of 20.6 mm (IQR: 15.1–27.2 mm)—corresponding to approximately two grid points (inter-grid distance ≈ 11.1 mm)—indicates that the conventional 10–20 EEG system may not capture individual anatomical variations in swallowing motor representation for a substantial proportion of patients.

In the most extreme case, the optimized position was displaced by 36.5 mm (approximately three grid points) from the conventional position, resulting in an EF increase of 0.13 V/m. Such displacements suggest that conventional fixed placement may result in suboptimal target engagement in patients whose individual anatomy diverges substantially from population-average scalp landmarks. However, the degree to which improved modeled EF translates to enhanced neurophysiological or clinical effects remains to be established in prospective studies.

Although the optimized anode was generally closer to the individualized cortical target than the conventional anode, the median reduction in target distance was small relative to the total electrode displacement. Moreover, the degree of target approximation was not significantly associated with the magnitude of EF gain. Most notably, target EF strength increased in every patient whose optimized anode was positioned farther from the target than the conventional anode, and the magnitude of improvement did not differ significantly according to whether the anode moved closer to or farther from the target. These findings indicate that the benefit of individualized optimization appears to arise primarily from reconfiguration of the overall current pathway through the head rather than from simply positioning the anode closer to the cortical target.

In the unadjusted analyses, lesion volume was not significantly correlated with the Opt-C–Conv-C change in EF strength (*r_s_* = −0.173), the corresponding change in target-specific focality (*r_s_* = 0.096), or the displacement between the optimized and conventional anodes (*r_s_* = −0.122). By contrast, in the mixed-effects model, larger lesion volume was associated with a greater target-specific focality across montages, while the montage-by-lesion-volume interaction was not significant. Taken together, these findings suggest that lesion volume may be related to the overall level of suprathreshold target engagement but does not explain the relative gain achieved by anode optimization. Gross lesion volume alone is therefore unlikely to capture spatial determinants of current flow, such as lesion proximity, geometry, and orientation relative to the current path. Previous computational work (47) demonstrated that lesion proximity to the target region and orientation relative to the predominant direction of current flow significantly affected EF distribution. Given that our optimized montage targets the unaffected hemisphere, the lack of correlation with lesion volume may reflect the relatively distal relationship between the lesion and the stimulated contralesional cortex. Nevertheless, lesion location, perilesional CSF replacement, and target-to-lesion geometry may provide more informative predictors of EF variability than volume alone, and future studies should investigate these factors in greater detail.

From a methodological standpoint, a critical prerequisite for reliable EF modeling in stroke populations is accurate segmentation of affected tissue, whose electrical conductivity differs substantially from that of healthy brain tissue. Unlike conventional simulation pipelines that either omit lesion segmentation or model lesions as simplified homogeneous regions, our approach—utilizing tES LAB 4.0’s deep neural network–based segmentation module—generates individualized head models that incorporate the altered electrical properties of affected tissue (20). By integrating stroke-specific anatomical information directly into the FEM simulation, our pipeline enables more biophysically realistic prediction of EF distributions than methods relying on lesion-naïve head models.

Furthermore, whereas prior computational tDCS studies, including those using SimNIBS (33), have often assessed stimulation coverage using whole-brain focality metrics that quantify suprathreshold tissue volume irrespective of anatomical specificity, such global measures may fail to capture stimulation precision within small, functionally defined targets such as the CSA. The target-specific focality metric adopted in this study quantifies the concentration of high-intensity EFs within a predefined swallowing motor ROI and directly corresponds to the prespecified ROI-based optimization objective of the present study. These whole-brain and single-ROI measures address different spatial scales, but neither directly quantifies EF engagement at the network level. Network-level EF quantification may be particularly relevant in stroke because swallowing is supported by a distributed network and lesions may alter its topology or disconnect specific nodes. The anatomy-informed framework described by Stoupis and Assecondi (48) provides a methodological example of this complementary level of analysis. Because network-level EF delivery was not evaluated in the present study, future studies incorporating swallowing-network analyses may help determine whether ROI-based optimization also improves EF distribution across the broader network.

Several limitations should be considered. The target and head models were necessarily simplified. The CSA hotspot was approximated as a 2-mm-radius spherical ROI, which did not capture the broader swallowing network, including the supplementary motor area, cerebellum, and brainstem. Accordingly, maximizing EF within the selected CSA ROI should not be interpreted as demonstrating optimized EF delivery across the distributed swallowing network. Nevertheless, centering the ROI on the fMRI-defined overlap of the mylohyoid and pharyngeal representations provided a functionally relevant anchor for optimization of the primary swallowing motor cortex. Although age-related brain atrophy was accounted for, the models did not incorporate white matter lesions, periventricular changes, or lesion-specific conductivity variations, all of which may alter current flow and reduce EF strength. Future models incorporating more refined measures of these changes (49) may improve the precision of individualized montage design. In addition, DTI-derived anisotropic conductivity could be incorporated to represent the directional organization of white matter in the FEM, potentially improving the reliability and personalization of predicted EF distributions (50). High-resolution three-dimensional T1-weighted acquisitions of the quality required for individualized head modeling are not routinely available in all clinical settings, which may limit the immediate implementation of this workflow. Further research is therefore needed to develop and validate image-preprocessing methods, such as resolution enhancement, that could enable reliable tissue segmentation and EF modeling using lower-resolution MRI obtained as part of routine clinical care.

Lesion characterization was also limited to volume, side, and a cortical, subcortical, or combined classification. Because lesion masks and target coordinates were unavailable in a common anatomical space, minimum lesion-boundary-to-target distance, lesion-centroid-to-target distance, and lesion–target overlap could not be calculated. Thus, despite no detected montage interactions with the measured lesion characteristics, effect modification by lesion proximity, geometry, or orientation relative to current flow cannot be excluded, particularly given the three patients with combined lesions. Addressing this question will require prospectively retained, spatially registered lesion masks. Electrode displacement was defined as the straight-line three-dimensional Euclidean distance between optimized and conventional anode centers, rather than a measure of lesion proximity, and may underestimate separation along curved scalp or cortical surfaces. The whole-brain EF maximum was not extracted; therefore, we could not confirm that the field maximum coincided with the target.

The findings also remain limited to computational target engagement. As an in silico study, we could not determine whether improvements in EF strength and target-specific focality translate into enhanced corticospinal excitability (51) or clinical swallowing outcomes. The present study was intended to establish a biophysical rationale for precision targeting in PSD; the clinical effects of the same MRI-guided individualized approach are being evaluated in the randomized trial described above (21). In addition, fixing the supraorbital cathode restricted the analysis to montage-specific anode optimization rather than the full two-electrode parameter space. This constraint followed the unilateral montage used in the swallowing-tDCS and stroke literature and isolated the effect of anode position, but simultaneous anode and cathode optimization remains to be tested.

Finally, the montage comparisons and outcome measure require cautious interpretation. Although small circular electrodes improved EF parameters relative to large square electrodes, this comparison jointly varied electrode size and shape, and the optimized two-electrode montage was not directly compared with HD-tDCS. Whether it can provide comparable focality with greater operational simplicity remains unknown. EF strength was calculated as the mean L2 norm within the target ROI using the untrimmed EF distribution, without excluding values above the 99th percentile. Although the same procedure was applied to all montages, the robustness of the results to localized extreme values was not evaluated using a percentile-trimmed sensitivity analysis. Target-specific focality was defined as the target-ROI volume exceeding the 75th percentile of EF values across GM and WM. This measure captures relatively high-intensity target engagement but does not quantify whole-brain off-target spread, and its relationship to therapeutic benefit and off-target effects requires validation.

## 5 Conclusion

In conclusion, the results from computational modeling demonstrate that personalized MRI-based computational optimization can substantially improve tDCS targeting of the CSA in stroke patients compared with conventional montage design based on fixed scalp coordinates. By integrating a stroke-specific modeling framework—including individualized lesion segmentation—with small circular electrodes and a target-specific focality metric, we provide a practical strategy for precision neuromodulation in PSD. These findings provide a computational basis for future clinical trials to determine whether individualized stimulation can improve swallowing recovery and reduce dysphagia-related complications after stroke.

## Data Availability

The raw data supporting the conclusions of this article will be made available by the authors, without undue reservation.
