# Supplementary material for: MRI-guided personalized tDCS planning for target engagement of the swallowing motor cortex after stroke: a finite-element modeling study
Source: Front Neurol. 2026 Sep 17;17:1898885. doi: 10.3389/fneur.2026.1898885 (PMC13627350; doi:10.3389/fneur.2026.1898885)
Supplement: Supplementary file 1 [file Image_1.PDF]

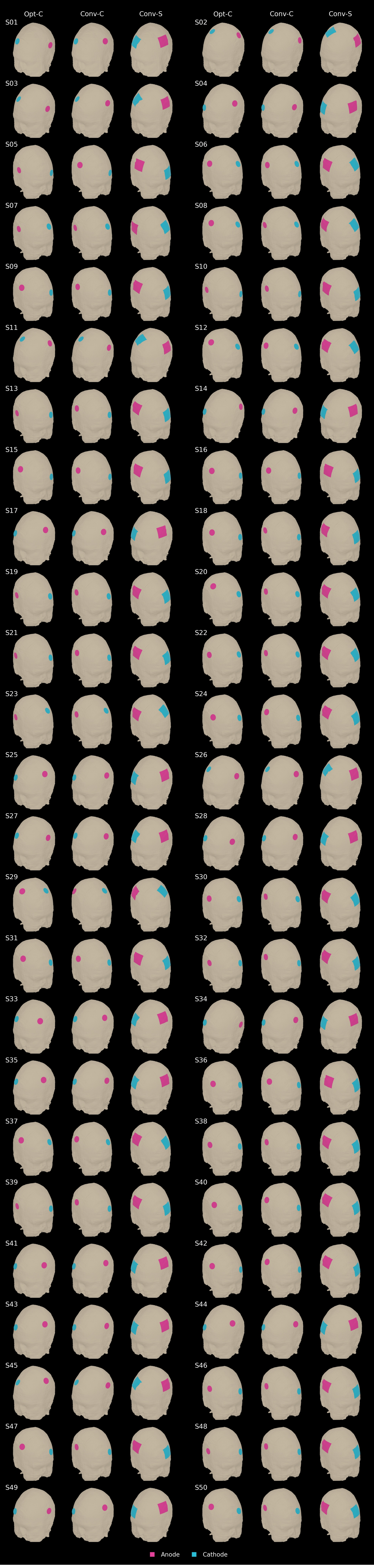

Figure S1. Patient- and condition-specific electrode montages on the reference head model. Electrode montages for all 50 patients (S01–S50) are shown for each condition in the MNI space of the reference head model.
