# Supplementary material for: MRI-guided personalized tDCS planning for target engagement of the swallowing motor cortex after stroke: a finite-element modeling study
Source: Front Neurol. 2026 Sep 17;17:1898885. doi: 10.3389/fneur.2026.1898885 (PMC13627350; doi:10.3389/fneur.2026.1898885)
Supplement: Supplementary file 2 [file Table_1.DOCX]

Supplementary Table 1. Manufacturer-specific MRI acquisition parameters recommended for tES LAB 4.0

| Type | GE Healthcare | Philips Medical Systems | Siemens Healthineers |
| --- | --- | --- | --- |
| Slice thickness (mm) | 1.2 | 1.2 | 0.9-1.2 |
| TR (ms) | 6-8 | 6-9 | 1800-2300 |
| TE (ms) | 2.5-3.5 | 3-4.5 | 2-3 |
| TI (ms) | 400-900 | 0 | 900 |
| Magnetic field strength (T) | 3 | 3 | 3 |
| Flip angle (°) | 8-11 | 8-9 | 9 |
| Acquisition matrix | 256 × 256 | 256 × 256 | 256 × 256 |
| Acquisition type | 3D | 3D | 3D |
| Number of slices | 192 | 170 | 176–192 |

Values represent recommended reference parameters rather than mandatory acceptance limits; scanner- and sequence-specific variations may be acceptable provided that the images satisfy the core structural input and image-quality requirements.
